# αCharges: partial atomic charges for AlphaFold structures in high quality

**DOI:** 10.1093/nar/gkad349

**Published:** 2023-05-09

**Authors:** Ondřej Schindler, Karel Berka, Alessio Cantara, Aleš Křenek, Dominik Tichý, Tomáš Raček, Radka Svobodová

**Affiliations:** CEITEC – Central European Institute of Technology, Masaryk University, 625 00 Brno, Czech Republic; National Centre for Biomolecular Research, Faculty of Science, Masaryk University, 625 00 Brno, Czech Republic; Department of Physical Chemistry, Faculty of Science, Palacký University Olomouc, 779 00 Olomouc, Czech Republic; CEITEC – Central European Institute of Technology, Masaryk University, 625 00 Brno, Czech Republic; National Centre for Biomolecular Research, Faculty of Science, Masaryk University, 625 00 Brno, Czech Republic; Institute of Computer Science, Masaryk University, 602 00 Brno, Czech Republic; Faculty of Informatics, Masaryk University, 602 00 Brno, Czech Republic; CEITEC – Central European Institute of Technology, Masaryk University, 625 00 Brno, Czech Republic; National Centre for Biomolecular Research, Faculty of Science, Masaryk University, 625 00 Brno, Czech Republic; CEITEC – Central European Institute of Technology, Masaryk University, 625 00 Brno, Czech Republic; National Centre for Biomolecular Research, Faculty of Science, Masaryk University, 625 00 Brno, Czech Republic

## Abstract

The AlphaFold2 prediction algorithm opened up the possibility of exploring proteins’ structural space at an unprecedented scale. Currently, >200 million protein structures predicted by this approach are deposited in AlphaFoldDB, covering entire proteomes of multiple organisms, including humans. Predicted structures are, however, stored without detailed functional annotations describing their chemical behaviour. Partial atomic charges, which map electron distribution over a molecule and provide a clue to its chemical reactivity, are an important example of such data. We introduce the web application αCharges: a tool for the quick calculation of partial atomic charges for protein structures from AlphaFoldDB. The charges are calculated by the recent empirical method SQE+qp, parameterised for this class of molecules using robust quantum mechanics charges (B3LYP/6-31G*/NPA) on PROPKA3 protonated structures. The computed partial atomic charges can be downloaded in common data formats or visualised via the powerful Mol* viewer. The αCharges application is freely available at https://alphacharges.ncbr.muni.cz with no login requirement.

## Graphical Abstract

**Graphical Abstract:**
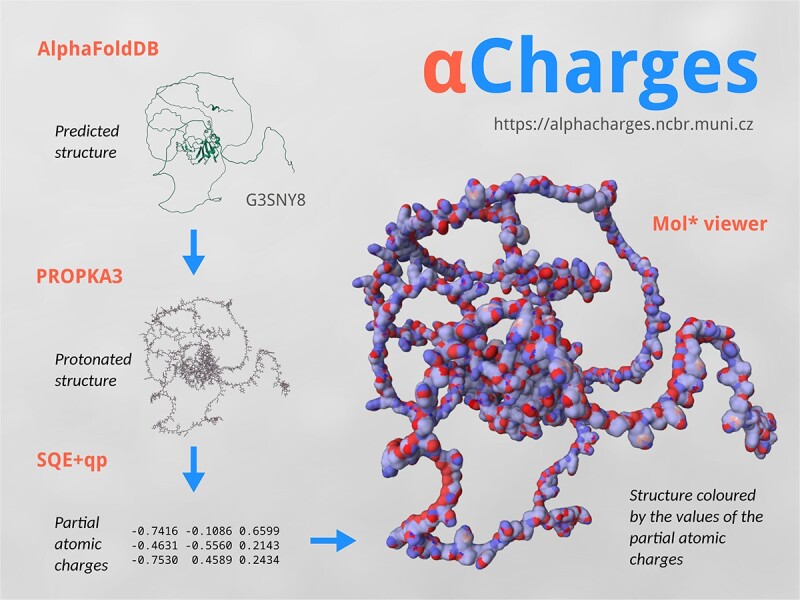
A schema describing the 𝛂Charges workflow: A structure is obtained from AlphaFoldDB and protonated by PROPKA3. Its charges are calculated by SQE+qp and visualized with Mol*.

## INTRODUCTION

The AlphaFold2 prediction algorithm (1) provided us with markedly greater insight into the structural space of proteins. Specifically, the set of 200 000 experimentally determined protein structures in the Protein Data Bank (2) is currently extended by >200 million predicted protein structures in AlphaFoldDB (3). This enables the research community to consider and hypothesise on scientific questions that could not have been touched until now.

It is beneficial to enrich the predicted protein structures with properties describing their chemical behaviour to support this research. A typical example of such data is partial atomic charges (4), which map the electron distribution over the molecule and provide a clue to its chemical reactivity. Partial atomic charges enable us to compare binding sites, including their polarity (5), identify hydrophobic membrane parts (4), or drive protein-protein interactions (6). They are also used as inputs for molecular docking (7), molecular dynamics (8), and other simulations (9,10).

The most reliable way to compute partial atomic charges is using quantum mechanics (QM) (11), which can provide electron density distribution among molecular orbitals. Afterwards, its mapping to atoms can be calculated by population analyses, e.g. Natural population analysis (NPA) (12,13). Unfortunately, QM methods are very time-consuming, and therefore only applicable to small molecules.

A faster alternative is the usage of empirical charge calculation methods (4), which mimic QM approaches and are based on significantly less complex empirical equation systems describing the charge distribution. These empirical methods are parameterised using QM charges calculated by a certain combination of a QM method and a population analysis. Charges computed by the empirical method mimic QM charges used in the parameterisation process. Even though the empirical methods are fast, it is challenging to apply them to proteins. One reason is that proteins are very large systems, and the empirical methods must be optimised to handle them. Moreover, proteins are homogeneous (i.e. they contain atoms with similar neighbourhoods), complicating the parameterisation of empirical methods. For these reasons, empirical charge calculation methods applicable to proteins have been developed in the last several years. We developed the SQE+qp method (14), which is currently the best-performing approach for proteins. We are also the authors of ACC II (4), a web server that provides charge calculation for almost any structure using 20 empirical methods. ACC II also includes the SQE+qp approach.

A limitation of ACC II is that it requires a protonated protein structure. Additionally, it implicitly deals with electroneutral molecules (i.e. molecules that have a zero total charge). The total charge is computed as a sum of the formal charges of individual atoms specified in the input file. A structure with a non-zero total charge can be provided. However, the user has to compute it by him or herself and include it in the input file. Moreover, the empirical methods in ACC II are only parameterised for electroneutral molecules. On the other hand, protein structures produced by the AlphaFold2 algorithm and contained in AlphaFoldDB do not include hydrogens, and after protonation, most of them are not electroneutral.

For this reason, we introduce αCharges, which solves all these challenges and provides the following functionality:

- The input protein structure from AlphaFoldDB is protonated using PROPKA3 (15,16) based on the pH value provided by the user (physiological pH is used as the default value).
- After protonation, the total charge of the protein is computed and used as an input for the SQE+qp method.
- The SQE+qp method is parameterised directly for protein-like structures.
- αCharges integrates the Mol* viewer (17), a cutting-edge molecular visualiser also used in AlphaFoldDB (3), PDBe (2) and RCSB PDB (18).

Therefore, αCharges provides high-quality partial atomic charges for AlphaFoldDB structures in one click. The user only has to provide the UniProt ID of the protein, and no additional knowledge or preprocessing is required.

## DESCRIPTION OF THE WEB SERVER

The application back end is written in Python, using the Flask web framework (https://flask.palletsprojects.com/). The front end is built with the Bootstrap library (https://getbootstrap.com/).

### SQE+qp method

The computation of partial atomic charges is carried out according to the SQE+qp model (14), which belongs to the family of electronegativity equalisation methods, like the popular EEM (19), QEq (8) or SQE (20).

As SQE added bond information to the EEM formalism, SQE+qp further improved it by adding a parameterised initial charge for each atomic type. In this context, an atomic type represents a class of atoms with similar characteristics (e.g. all carbon atoms with only single bonds or all hydrogen atoms bound to oxygen atoms).

These initial charges and other model parameters have to be chosen appropriately for the given class of molecules (e.g. peptides, drug-like molecules, nucleic acids) to compute high-quality partial atomic charges.

### αCharges workflow

**Figure 1.**
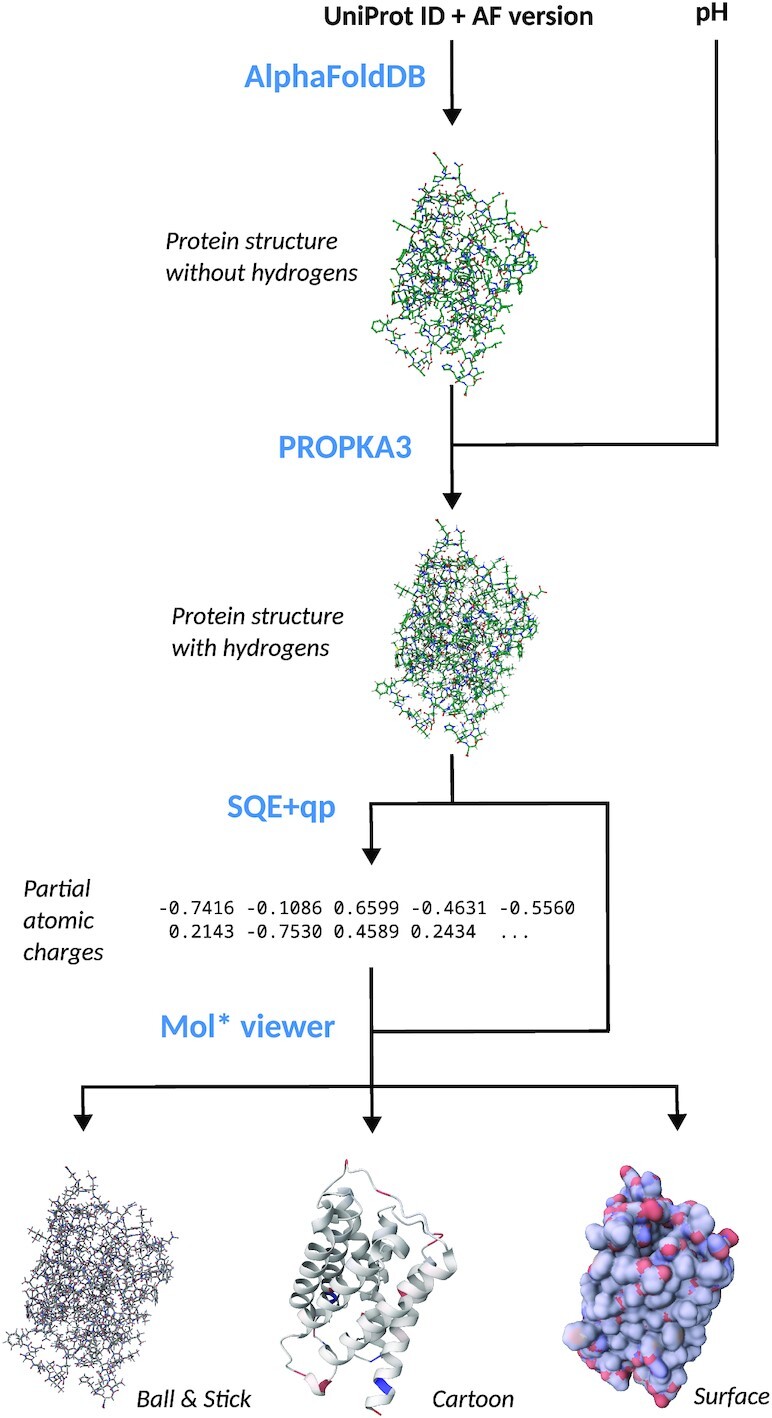
Schematic depiction of αCharges workflow. The schema includes a structure of porin (UniProt ID: A0A2E5IBK2) originating from *Euryarchaeota archaeon*.

One of the major design decisions for αCharges was to provide the user with an intuitive interface that would facilitate the calculation of partial atomic charges without needing to configure all the parameters of the process. The workflow is depicted in Figure 1 and can be described in several steps:

- *Downloading the structure from AlphaFoldDB API:* The first step is to enter the UniProt ID (or AlphaFoldDB ID) of a selected protein structure into the textbox on the landing page. The structure is downloaded in PDB format if the ID is valid. Currently, structures are downloaded in the latest version (i.e. AlphaFoldDB version 4, as of February 2023). However, if a user clicks the *Setup calculation* button, any prior version can be selected.
- *Addition of hydrogens by PDB2PQR/PROPKA3:* Once the structure is downloaded, adding hydrogen atoms is the next step. For this purpose, αCharges uses PDB2PQR (21) with the PROPKA3 library (15,16). By default, the protonation is done using the physiological pH of 7.2. However, any value between 0 and 14 can be specified on the *Setup* page.
- *Calculation of charges by SQE+qp:* If the protonation succeeds, partial atomic charges can be computed. Since the input structure might be large (i.e. tens of thousands of atoms), even quite effective empirical methods such as SQE+qp might be too memory-demanding and slow, since they have a cubic computational complexity with respect to the number of bonds in the molecule.
  To overcome the issue of computation complexity, αCharges uses the Cutoff/Cover approach (22), which first divides the molecule into smaller regions (spheres centred around individual residues). Then, the computation is carried out independently for each region. In the next step, the partial atomic charges are corrected so that their sum equals the desired total molecular charge. First, the correction is computed for each small region. Then, the charges of the whole structure are revised in the same way.
  The quality of the parameters for the empirical charge calculation method heavily influences the quality of the resulting charges. αCharges uses parameters fitted to reproduce B3LYP/6-31G*/NPA charges originally parameterised on the set of peptides and later optimised for proteins. Charges computed using these parameters were shown to achieve an excellent agreement with the reference charges (14).
- Visualisation of the charges with Mol*: After calculating partial atomic charges, the user is redirected to the Results page, whose central part is Mol* viewer (17). Mol* was extended to support the visualisation of partial atomic charges at several levels of protein structure depending on the chosen view. The default *cartoon* representation colours each residue by the sum of the charges of its atoms. The two other visualisation modes are *ball & stick* and *surface*.
  The values of charges are mapped onto a linear blue-white-red gradient, where blue means a positive charge, white signifies zero charge, and red equals a negative charge. Two main colouring types are available in the application. In the default *relative colouring*, the gradient is mapped to the charges such that the highest and lowest charges are represented by the most saturated shades of blue and red, respectively. If a user wants to generate several images of various structures to compare the charge distribution, *absolute colouring* can be used. In this case, the maximum value of positive and negative charge can be configured manually.
- *Download of computed charges:* Although the visual representation of the charges is undoubtedly valuable for humans, the charges can also be downloaded if further processing is required. αCharges includes files of several formats in a downloaded ZIP file for user convenience. These include:
  - PDB file with added hydrogens
  - PQR file with added hydrogens and calculated charges
  - mmCIF file with added hydrogens and calculated charges
  - a simple TXT file with calculated charges

### Limitations

αCharges cannot compute partial atomic charges for proteins that contain structural errors because of an incorrect AlphaFold2 prediction. Furthermore, PROPKA3 is unable to protonate some structures correctly. In these cases, an appropriate error message is given to the user. The message lists all atoms that were identified as problematic. After clicking on their names, they are highlighted in Mol* so the user can visually inspect the issues.

## RESULTS AND DISCUSSION

We put the application through extensive testing to confirm that it is stable and reliable. Details of this testing can be found in the Supplementary Data.

Furthermore, we provide three use cases demonstrating various applications of αCharges. They are presented interactively on the αCharges webpage.

### Example I: P-glycoprotein

**Figure 2.**
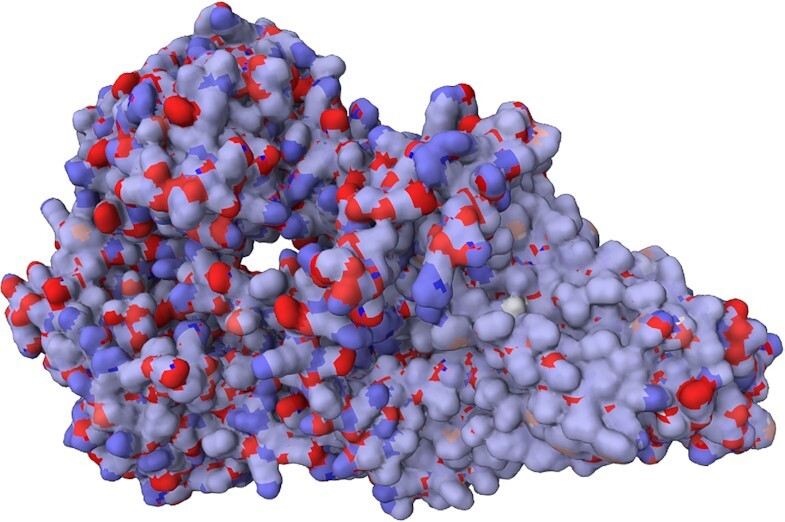
Structure of P-glycoprotein from *Caenorhabditis elegans* (UniProt ID: P34712). Partial atomic charges calculated by αCharges are visualised on the protein’s surface. They show distinct areas of the protein: The nonpolar transmembrane part (mostly very light blue due to the charge being around zero on the surface) and the polar surface of extracellular and cytoplasmic parts (with a mosaic of blue positive and red negative charges).

P-glycoprotein is one of the ABC transporter proteins that decrease drug accumulation in cancer cells (23). It is a 170kDa protein consisting of a nucleotide-binding domain and a transmembrane domain (24). We used a P-glycoprotein structure model from *Caenorhabditis elegans* (UniProt ID P34712). Visualising the charges (Figure 2) computed by αCharges demonstrates the differences in charge distribution between the transmembrane parts and extracellular/intracellular ones.

### Example II: pepsin

**Figure 3.**
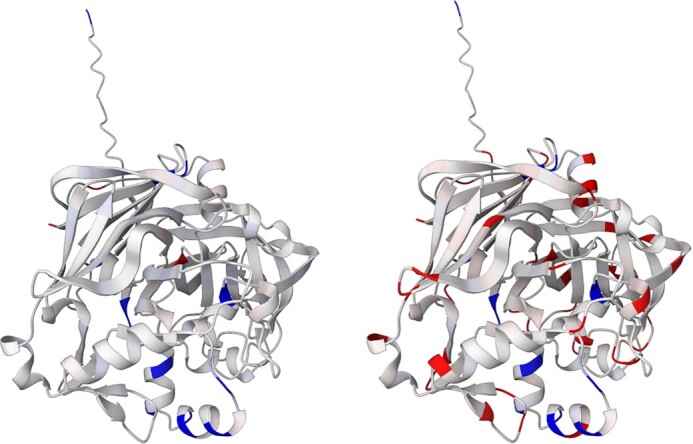
Structure of pepsin (UniProt ID: P00791), partial atomic charges calculated by αCharges are visualised on the cartoon model of the protein showing significant charge differences between an active form (left image, pH 2) and an inactive form (right image, pH 7).

Pepsin is an enzyme that plays a major role in protein digestion in the stomach. It is secreted as a zymogen and activated by the acidic pH created by the stomach parietal cells. Pepsin is most effective at a pH of approximately 1.5 to 2, and becomes inactive when the pH rises above 6 (25). The enzyme denatures at a pH 8 (26). Differences in the charge distribution between its active form (pH 2) and inactive form (pH 7) can be seen in this use case (Figure 3). The alkaline environment causes an increase in negative charges, which contributes to the structural instability of the pepsin, causing the shift to an inactive form of the protein (27,28).

### Example III: PIN proteins

The PIN family proteins control plant growth by regulating auxin export from the cytosol to the extracellular space (29). Eight types of PIN transport proteins are known (PIN1 to PIN8) and they are divided into two classes: canonical PINs (PIN1-4 and PIN7) and non-canonical PINs (PIN5, PIN6 and PIN8) (29). This year, structures of three of them were discovered and published (PIN1 (30), PIN3 (31) and PIN8 (29)), while structures of the remaining ones are waiting to be revealed. But thanks to AlphaFoldDB, we can see their predicted structure and compare their charge distribution.

**Figure 4.**
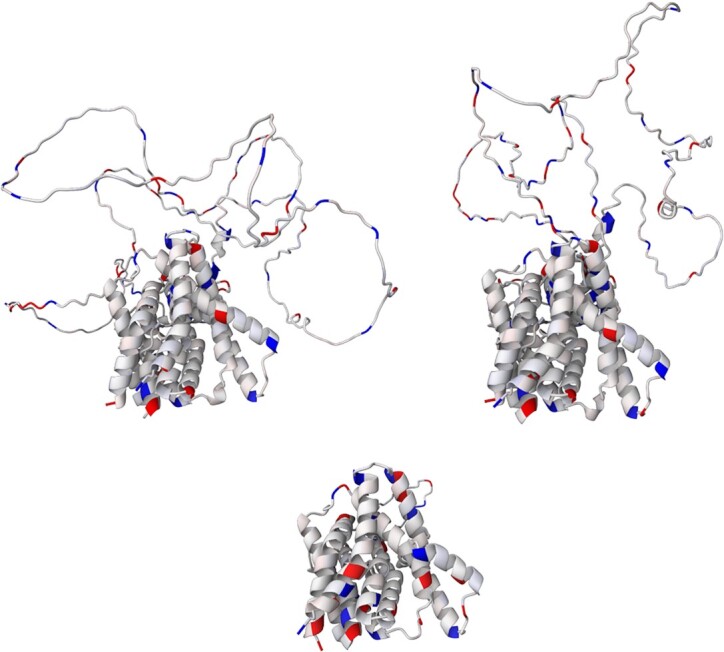
Structure of PIN3 (top left image, UniProt ID: Q9S7Z8), PIN7 (top right, UniProt ID: Q940Y5) and PIN5 (bottom image, UniProt ID: Q9FFD0) from *A. thaliana*. Partial atomic charges calculated by αCharges are visualised in a cartoon model.

Charge distribution of canonical PINs is very similar, even though sequence and structure strongly differ. It can be seen especially when we compare PIN3 and PIN7 (see Figure 4, top images). On the other hand, charge distribution of non-canonical PINs differs from each other and also from canonical PINs (see PIN5 in Figure 4, bottom image).

## CONCLUSION

In this article, we presented αCharges, a novel web application for calculating partial atomic charges on protein structures produced by the AlphaFold2 algorithm and available in AlphaFoldDB. αCharges utilises the SQE+qp empirical charge calculation method, parameterised using B3LYP/6-31G*/NPA quantum mechanical charges. αCharges allows users to download charges (in PQR, mmCIF, or plaintext formats) or visualise them via three main structure visualisation models (cartoon, ball & stick, and surface). The web application is easy to use and is platform-independent. Documentation explaining the usage of the tool is provided on the webpage.

## DATA AVAILABILITY

αCharges application is freely available at https://alphacharges.ncbr.muni.cz (hosted by the highly available Masaryk University computing cloud) with no login requirement. The user manual for the application is available at https://github.com/sb-ncbr/AlphaCharges/wiki, while the source code is accessible at GitHub under the MIT licence at https://github.com/sb-ncbr/AlphaCharges/ and is also available in the Supplementary Data. The code is also available on Zenodo at https://doi.org/10.5281/zenodo.7844436.

## Supplementary Material

gkad349_Supplemental_Files
