# Supplementary material for: αCharges: partial atomic charges for AlphaFold structures in high quality
Source: Nucleic Acids Res. 2023 May 9;51(W1):W11–6. doi: 10.1093/nar/gkad349 (PMC10320090; doi:10.1093/nar/gkad349)
Supplement: gkad349_Supplemental_Files [file gkad349_supplemental_files.zip › supplementary_testing.pdf]

Service performance and stability

We stress-tested the service with parallel requests for computing 9,984 randomly selected Uniprot ID samples from more than 200 million proteins in AlphaFoldDB. Each sample was included in the test in AlphaFold prediction versions 3 and 4. Every included sample was protonated with 8 different pH levels in the range of 4.5 to 8. This setup yields a total of 159,744 requests.

For this test, the service was run on a 16-core AMD Ryzen 9 7950X machine and was configured to support 32 parallel requests. 20 clients were run in parallel, each submitting its requests at the maximum possible speed (i.e. sending a new request immediately after receiving a response to the previous one). During the test, the machine exhibited 70 to 90 % CPU utilisation, i.e. it was not fully saturated. We measured whole-request times, covering all the request stages, including the structure download.

413 requests (0.26 %) failed on the client side due to network errors. We exclude those from the statistic. Furthermore, 6,132 requests (3.8 %) failed due to a PROPKA3 failure, which can sometimes be caused by unreliable AlphaFold prediction. We observed long sections of proteins without secondary structure when inspecting some of the AlphaFoldDB entries with failed computation, e.g. A0A452I6S5). This mostly occurs when an extreme, non-physiological pH is requested (e.g. protonation of A0A2D1ISF4 fails with pH 4.5 but succeeds at the default pH value of 7.2). Surprisingly, the number of failures was similar for both versions of the AlphaFold prediction (3,367 for version 3 versus 2,765 for version 4).

We observed a mean time for the successful completion of requests of 11.7 s, with a median of 9.0 s. The distribution of the request times tends to be exponential, with 92 % of the requests being below 20 % of the maximum time (131 s). However, we could not reproduce any of the long requests that took over 100 s. Repeated attempts to do so always fell below 30 s. We conclude, though without direct evidence, that the reason for the occurrence of requests with extreme completion time was communication with the AlphaFoldDB server that hit one of its random load peaks and was slow to respond.
