# Supplementary material for: αCharges: partial atomic charges for AlphaFold structures in high quality
Source: Nucleic Acids Res. 2023 May 9;51(W1):W11–6. doi: 10.1093/nar/gkad349 (PMC10320090; doi:10.1093/nar/gkad349)
Supplement: gkad349_Supplemental_Files [file gkad349_supplemental_files.zip › supplementary_application_sources.zip!/AlphaCharges-main/app/templates/404.html]

{% extends 'base/base.html' %}
{% block title %} AlphaCharges – Page not found {% endblock title %}
{% block body %}

# **αCharges**

**404**: This isn't the page you're looking for.

Back to main page

---

{% include 'base/elixir.html' %}

---

{% include 'base/footer.html' %}

{% endblock body %}
{% block scripts %}
{% endblock scripts %}
