# Supplementary material for: αCharges: partial atomic charges for AlphaFold structures in high quality
Source: Nucleic Acids Res. 2023 May 9;51(W1):W11–6. doi: 10.1093/nar/gkad349 (PMC10320090; doi:10.1093/nar/gkad349)
Supplement: gkad349_Supplemental_Files [file gkad349_supplemental_files.zip › supplementary_application_sources.zip!/AlphaCharges-main/app/templates/base/elixir.html]

****αCharges** tool is
a
part
of services provided by
ELIXIR
–
European research infrastructure for biological information.
  
For other services provided by ELIXIR's Czech Republic Node visit
www.elixir-czech.cz/services.**

---

Licence conditions in accordance with § 11 of Act No. 130/2002 Coll. The owner of the software is Masaryk
University, a public university, ID: 00216224. Masaryk University allows other companies and individuals to
use this software free of charge and without territorial restrictions in usual way, that does not depreciate
its value. This permission is granted for the duration of property rights. This software is not subject to
special information treatment according to Act No. 412/2005 Coll., as amended. In case that a person who
will use the software under this licence offer violates the licence terms, the permission to use the
software terminates.
