# Supplementary material for: αCharges: partial atomic charges for AlphaFold structures in high quality
Source: Nucleic Acids Res. 2023 May 9;51(W1):W11–6. doi: 10.1093/nar/gkad349 (PMC10320090; doi:10.1093/nar/gkad349)
Supplement: gkad349_Supplemental_Files [file gkad349_supplemental_files.zip › supplementary_application_sources.zip!/AlphaCharges-main/app/templates/base/flash.html]

{% with messages = get\_flashed\_messages(with\_categories=true) %}
{% if messages %}
{% for category, message in messages %}

×


{{ message }}

{% endfor %}
{% endif %}
{% endwith %}
