# Supplementary material for: αCharges: partial atomic charges for AlphaFold structures in high quality
Source: Nucleic Acids Res. 2023 May 9;51(W1):W11–6. doi: 10.1093/nar/gkad349 (PMC10320090; doi:10.1093/nar/gkad349)
Supplement: gkad349_Supplemental_Files [file gkad349_supplemental_files.zip › supplementary_application_sources.zip!/AlphaCharges-main/app/templates/computation_progress.html]

{% extends 'base/base.html' %}
{% block title %} αCharges – Calculation results {% endblock title %}
{% block styles %}
{% endblock styles %}
{% block body %}

# **αCharges – Calculation progress**

**UniProt code:**  {{ code }}

**AlphaFold2 prediction version:** {{ alphafold\_prediction\_version }}

**pH:** {{ ph }}

---

Loading...

{% endblock body %}
{% block scripts %}

{% endblock scripts %}
