# Supplementary material for: αCharges: partial atomic charges for AlphaFold structures in high quality
Source: Nucleic Acids Res. 2023 May 9;51(W1):W11–6. doi: 10.1093/nar/gkad349 (PMC10320090; doi:10.1093/nar/gkad349)
Supplement: gkad349_Supplemental_Files [file gkad349_supplemental_files.zip › supplementary_application_sources.zip!/AlphaCharges-main/app/templates/index.html]

{% extends 'base/base.html' %}
{% block body %}

# **αCharges**

**AlphaCharges** (**αCharges**)
is a web application for the calculation of partial atomic charges on protein structures predicted by
the AlphaFold2 algorithm and deposited in the AlphaFoldDB database. The charges are computed by the SQE+qp empirical method, which quality is comparable to quantum mechanical
charge calculation approaches (specifically, it is parameterized using B3LYP/6-31G\*/NPA quantum
mechanical charges). Before computation of the charges, **αCharges** protonates
the
input protein structures by PROPKA3. The details about the methodology and usage are
described in the manual. This website is free and open to all users and there is no login
requirement. Source codes are freely available at GitHub.

---

{% include 'base/flash.html' %}
  


**UniProt code:**

Calculate
charges
Setup calculation


---

### Examples

**P-glycoprotein**

P-glycoprotein is part of the ABC transporter proteins that decrease drug accumulation in cancer
cells (Leslie2005).
It is a 170-kDa protein which consists of a nucleotide-binding domain and a transmembrane domain
(Ward2013).
Partial atomic charges calculated by
**αCharges**
demonstrate the differences in charge
distribution between transmembrane parts and the extracellular/intracellular one.

P-glycoprotein

**Pepsin**

pH: 1

Pepsin is the enzyme that is majorly involved in protein digestion in the stomach. It is secreted
as a zymogen and activated by the acidic pH created by the stomach parietal cells. Pepsin is the
most effective at a pH of approximately 1.5 to 2, and it becomes inactive when the pH rises above 6
(Heda2022). Pepsin remains structurally stable until at least a pH of 8.
Differences in a charge distribution between its active form (pH 2) and inactive form (pH 8) can be
seen in this use case. The alkaline environment causes an increase in negative charges, which
contributes to the structural instability of the pepsin, causing the shift to an inactive form of
the protein (Tanaka2001, Grahame2021).

Active


Inactive

**PIN proteins**

The PIN family proteins control plant growth by regulating auxin export from the cytosol to the
extracellular space. Eight types of PIN proteins are known (PIN1-PIN8), and last year, structures of
three of them were discovered and published in Nature (i.e., articles about 
PIN1,
PIN3,
PIN8).
Partial atomic charges play an important role in PINs functionality.
The PIN protein part inside the cytosol (containing the cytosolic loop) is charged more than the
part outside the cytosol. Questionable is the charge distribution of PIN5, which structure differs
from other PINs (Ung2022) and was not experimentally determined yet. In the
use case, you can compare the charge distribution of AlphaFold2 predicted PINs from *A.
thaliana*.

PIN1


PIN3


PIN5

PIN6


PIN7


PIN8

---

If you found
**αCharges** helpful,
please refer this web page, the
manuscript
is in preparation. Are you interested in a research collaboration? Feel free to contact us.

---

{% include 'base/elixir.html' %}

---

{% include 'base/footer.html' %}

{% endblock body %}
{% block scripts %}
{% endblock scripts %}
